# Dietary evaluation of *Pleurotus ostreatus* spent mushroom substrate on productivity, nutrient digestibility, egg quality, antioxidant status, and ovarian cytoprotective responses in laying hens

**DOI:** 10.1016/j.psj.2026.106623

**Published:** 2026-02-12

**Authors:** Ioannis Brouklogiannis, Georgios Koutrotsios, Georgios I. Zervakis, Konstantinos C. Mountzouris

**Affiliations:** a Laboratory of Nutritional Physiology and Feeding, Department of Animal Science, School of Animal Biosciences, Agricultural University of Athens, Iera Odos 75, 11855 Athens, Greece; b Laboratory of General and Agricultural Microbiology, Department of Crop Science, Agricultural University of Athens, 11855 Athens, Greece; c Feed Innovations & Technologies P.C., a spin-off company of the Agricultural University of Athens, Matsa 10, 14665, Kifisia, Greece

**Keywords:** Poultry, Oyster mushroom, β-glucans, Phenolic compounds, Sustainability

## Abstract

This study evaluated the dietary inclusion level effects of *Pleurotus ostreatus* spent mushroom substrate (SMS) on performance, nutrient digestibility, egg quality and oxidative stability in laying hens. Additionally, key ovarian homeostasis-related genes involved in detoxification (aryl hydrocarbon receptor-AhR), antioxidant (nuclear factor erythroid 2–related factor 2-Nrf2), and inflammatory response (nuclear factor kappa B-NF-κB) were assessed. A total of 196 Hy-Line Brown hens, 51-weeks of age, were assigned to 4 treatments with 7 replicates of 7 hens each, receiving isocaloric, isonitrogenous and isofibrous diets containing 0 (S0), 25 (S25), 50 (S50), or 100 (S100) g SMS/kg. Performance responses were determined weekly throughout the 7-week experimental period and are reported as overall. At the end of week 7, eggs were collected for quality and oxidative stability determination, and ovarian samples were obtained for qPCR analysis. Increasing SMS inclusion level, improved overall laying rate and egg mass, with optimal (*P* ≤ 0.05) performance notable in the S100 compared to S0, and enhanced ether extract digestibility (*P* < 0.01). Egg oxidative stability was significantly improved by SMS inclusion, as reflected by reduced yolk malondialdehyde and egg-white protein carbonyl levels in fresh and stored eggs (*P* ≤ 0.001), with the lowest values consistently observed in the S100 treatment. The nutrigenomic analyses revealed that dietary SMS inclusion beneficially modulated (*P* ≤ 0.05) the majority of ovary-expressed genes related to detoxification (AhR pathway; 3/4 genes), antioxidant defense (Nrf2 pathway; 5/9 genes), and inflammatory response (NF-κB pathway; 8/13 genes), with the most pronounced differences observed in the S100 treatment compared with the control treatment. Conclusively, dietary SMS inclusion at 100 g/kg enhanced layer performance, improved ether extract digestibility, and increased egg oxidative stability. In addition, nutrigenomic analyses revealed that the observed improvements in performance and egg quality were supported by beneficial modulation of key homeostasis-related genes relevant to ovarian function and health.

## Introduction

Lignocellulosic biomass originating mainly from agricultural residues is one of the most abundant renewable resources and relatively inexpensive, with annual production estimated at nearly 180 billion metric tons (Güleç et al., 2024). Among the biological processes capable of transforming and valorizing this biomass is the cultivation of edible mushrooms that receives growing attention. More specifically, production of oyster mushrooms (*Pleurotus* spp.) has increased markedly, representing approximately 19% of total global production (Güleç et al., 2024; Royse et al., 2017; Segers et al., 2024). A current challenge associated with mushroom production is the residue left at the end of the cultivation process, known as spent mushroom substrate (**SMS**). Typically, 4–5 kg of SMS are produced for every kilogram of mushrooms harvested. Disposal of SMS through landfilling or incineration poses environmental and economic concerns, prompting interest in its valorization (Koutrotsios et al., 2014; Martín et al., 2023; Phan and Sabaratnam, 2012; Törős et al., 2024). In this respect, repurposing SMS as a bioactive and nutrient source in animal diets presents a sustainable strategy aligned with circular bioeconomy principles.

Beyond its relatively low value as a feed ingredient, SMS may contain various bioactive compounds originating from the fungal biomass and the partially degraded cultivation substrate, including among others phenolics, ergosterol, and β-glucans (Bekiaris et al., 2020; Koutrotsios et al., 2017). These biologically active molecules may confer additional cytoprotective potential that could influence performance, egg quality and health-related responses in poultry. Indeed, improved cytoprotection could be highly relevant for high-performing laying hens that due to elevated metabolic activity generate increased levels of reactive oxygen species (**ROS**), whereas late-phase hens are more susceptible to age-related ROS accumulation and oxidative stress (Durand et al., 2022; Eid et al., 2021). The latter accelerates the ovarian aging contributing to impaired productivity, health, and egg quality. Therefore, nutritional strategies that modulate antioxidant mechanisms or attenuate inflammatory signaling may contribute to ovarian homeostasis, improved robustness, productivity, and egg quality in intensive layer production (Brouklogiannis and Mountzouris, 2025; Yoshida et al., 2017; Zhang et al., 2022).

Research on mushroom-derived materials in laying hens has mainly focused on mushroom stem waste (Abdel-Wareth et al., 2025; Karageorgou et al., 2024; Stamps et al., 2025; Yang et al., 2021), fermented substrates (Kim et al., 2014; Yoshida et al., 2017), or whole mushroom meals (Hwang, 2012; Natsir and Wicaksono, 2020; Ogbe et al., 2009). To the best of our knowledge, only one study has examined the direct inclusion of SMS in laying hens, and it evaluated performance indicators and egg yolk cholesterol (Arjin et al., 2015). Much less is known about how SMS influences molecular homeostasis related-responses including cytoprotection and control of inflammation. Consequently, the mechanistic basis by which SMS may exert beneficial effects in laying hens remains largely unexplored.

Therefore, the objective of the present study was to investigate the dietary inclusion level effects of SMS on productive performance, nutrient digestibility, egg quality traits and antioxidant status, as well as ovarian gene expression of key homeostasis-associated pathways in mid-phase laying hens. In particular, via a nutrigenomic approach, the mRNA expression levels of multiple gene biomarkers involved in pathways related to cytoprotection (aryl hydrocarbon receptor; **AhR /** nuclear factor erythroid 2–related factor 2; **Nrf2**) and inflammatory response (nuclear factor kappa B; **NF-κB**) were assessed.

## Materials and methods

### Ethical approval

All practices regarding the care and use of animals and sampling procedures were under institutional and national guidelines and approved (Protocol No. 26/13052021) by the Bioethics Committee of the Agricultural University of Athens (**AUA**), Greece.

### Animals, housing, diets and experimental treatments

In total, 196 Hy-Line Brown laying hens were reared in the AUA laying hen facility for a 2-week adaptation period. At the end of the 50th week of age, layers were allotted into 4 dietary treatments with 7 replicates of 7 layers each, ensuring uniform body weight and comparable baseline performance. The main experimental trial was conducted from 51 to 57 weeks of age, corresponding to a 7-week experimental period. Four experimental diets were formulated by supplementing a basal corn–soybean meal diet with increasing levels of spent mushroom substrate (SMS), resulting in: a control diet without SMS (S0; 0 g/kg), and diets containing 25 g/kg (S25), 50 g/kg (S50), or 100 g/kg of diet (S100) of SMS.

The SMS, derived from *Pleurotus ostreatus* cultivation on a wheat-straw–based substrate, was obtained from a commercial mushroom production facility and oven-dried in AUA at 60°C for 48 h, decreasing moisture from ∼65% to ∼8% (w/w). All diets were formulated to be isocaloric, isonitrogenous, and isofibrous, and were provided in mash form to meet the recommendations of the Hy-Line Brown Management Guide. Wheat straw was included in the basal formulation and the increasing levels of SMS replaced corresponding portions of this wheat straw to maintain equal fiber content across treatments. The ingredient and chemical composition of the diets are presented in Table 1. Hens had *ad libitum* access to feed and water throughout the experimental period. The laying hens were housed in three-tier battery enriched cages under controlled environmental conditions and were maintained on a 16L:8D photoperiod, following the lighting program recommended in the Hy-Line Brown Management Guide. The chemical composition of the SMS used in this study (as fed) was as follows: 92.31% dry matter, 5.43% crude protein, 33.92% crude fiber, 0.54% ether extract, 9.85% ash and 7.30% acid-detergent lignin (**ADL**). The SMS contained a total phenolic content of 5000.23 mg/kg, determined using the Folin–Ciocalteu method (expressed as gallic acid equivalents), and an ergosterol concentration of 136.50 mg/kg, quantified by HPLC–DAD. Regarding the β-glucan content, SMS fungal biomass was quantified by fungal chitin hydrolysis into N-acetylglucosamine according Scotti et al. (2001), that was then measured colorimetrically according to Ride and Drysdale (1972). The fungal biomass content averaged 136.9 mg/g dried SMS. Subsequently, β-glucan content was determined on the isolated fungal biomass using a commercial β-glucan assay kit (Megazyme, Bray, Ireland) and was found to be 13.67% (w/w, dry matter basis), corresponding to 18.7 g β-glucans per kg SMS (dry matter basis).

**Table 1:** Ingredient and chemical composition of the experimental diets (%).

Ingredients %	S0^7^	S25	S50	S100
Corn grain	49.71	48.98	48.26	46.65
Soybean meal (44%)	20.88	20.89	20.89	21.08
Soy Protein concentrate^1^	1.00	1.00	1.00	1.00
Soya oil	3.50	3.50	3.50	3.50
Vegetable fat^2^	2.26	2.54	2.83	3.41
Sunflower, extr, 28%	2.75	2.75	2.75	2.75
Straw wheat	8.28	6.22	4.15	0.00
Spent mushroom substrate	0.00	2.50	5.00	10.00
Limestone	9.46	9.46	9.45	9.43
Monocal phosphate, HCL	1.07	1.07	1.07	1.07
Salt (NaCl)	0.29	0.29	0.29	0.29
Sodium bicarbonate	0.09	0.09	0.09	0.09
L-lysine-HCL	0.04	0.04	0.04	0.04
DL-methionine	0.24	0.24	0.25	0.25
L-threonine	0.03	0.03	0.03	0.03
Vitamin premix^3^	0.20	0.2	0.2	0.2
Mineral premix^4^	0.20	0.2	0.2	0.2
Chemical composition^5^	%			
AME_n_, (MJ/kg diet)	11.20	11.20	11.20	11.20
Dry matter	90.57	90.75	90.92	91.27
Crude protein	14.50 (14.69)	14.50 (14.60)	14.50 (14.58)	14.56 (14.74)
Ether extract	8.00 (7.84)	8.23 (7.91)	8.47 (8.38)	8.97 (8.67)
Crude fiber	6.50 (6.81)	6.50 (6.89)	6.50 (6.91)	6.50 (6.95)
Calcium	4.00	4.00	4.00	4.00
Available phosphorus	0.35	0.35	0.35	0.35
Lysine	0.78	0.78	0.78	0.78
TSAA^6^(methionine+cysteine)	0.73	0.73	0.73	0.73
Threonine	0.58	0.58	0.58	0.58

1 Soy Protein concentrate with 530 g crude protein/kg (Alpha Soy 530, Agilia Europe, Skjernvej 42, DK-6920, Videbaek, Denmark).

2 Lecithinised fat powder with 6% lecithin (nufat 99 L, Nuevo SA, Schimatari, Viotia, Greece).

3 Vitamin premix (Rovimix 13 Lay Basic, DSM) provided the following per kg of diet: vitamin A (retinyl acetate), 10,000 IU; vitamin D3 (cholecalciferol), 3,000 IU; vitamin E (dl-α-tocopheryl acetate), 30 IU; vitamin K_3_, 3 mg; vitamin B1, 2 mg; vitamin B2, 5 mg; vitamin B5 (pantothenic acid), 10 mg; vitamin B6 (pyridoxine), 4 mg; vitamin B12 (cobalamine), 0.015 mg; nicotinic acid, 30 mg; vitamin B7 (biotin), 100 mg; vitamin B9 (folic acid), 800 mg.

4 Mineral premix (Rovimix Layer M, DSM) provided the following per kg of diet: manganese, 120 mg; zinc, 70 mg; iron, 40 mg; copper, 8 mg; iodine, 1 mg; selenium, 0.2 mg and choline chloride, 400 mg.

5 The values in parentheses were analyzed, and others were calculated.

6 Total sulfuric amino acids.

7 Treatments: S0=0 g/kg, S25=25 g/kg, S50=50 g/kg and S100=100 g/kg of SMS supplementation in the hens’ diet.

### Laying productive performance

Throughout the study, the total number of eggs produced and total egg weight were recorded daily. The laying rate (hen-day egg production) was determined as the total number of eggs produced per replicate divided by the number of hens per replicate and the number of days in the period, expressed as a percentage. The average egg weight was determined as the total egg weight divided by the number of eggs produced in 7 days. Furthermore, feed intake was determined as the difference in feed offered and consumed on a weekly basis. Egg mass was calculated by multiplying the average egg weight by the laying rate. Feed conversion ratio was calculated as grams of feed intake per gram of egg mass. The data for the performance responses (i.e., laying rate, egg mass, feed intake and feed conversion ratio) were reported as overall (1-7th experimental week). Mortality data were recorded daily throughout the experimental period. Mortality was monitored and recorded twice daily throughout the experiment.

### Total tract apparent digestibility of nutrients

Total tract apparent digestibility was evaluated following the procedures described by (Paraskeuas et al. (2023), adapted to the conditions of the present study. The digestibility assessment was conducted during the last week of the main experimental period. Total tract apparent digestibility was evaluated on a cage basis, with each treatment represented by seven replicate cages. Hens remained in their assigned cages, which allowed for complete excreta collection from each replicate. Before sampling, a 4-day adaptation period to the diets and housing conditions was allowed. Excreta were then collected for three consecutive days. During the collection period, excreta from each cage were collected three times daily, pooled per cage, and stored at –20°C in sealed bags until analysis. Feed samples and pooled excreta samples from each cage were analyzed for dry matter, ash, ether extract and crude protein. Apparent total tract digestibility coefficients for each nutrient were calculated using the following formula:

Apparent total tract digestibility (%) = {[(nutrient intake, g) – (nutrient excreted, g)] / (nutrient intake, g)} × 100.

### Egg quality traits and antioxidant status

At the end of the 7th experimental week (i.e., 57 weeks of layers’ age), 3 eggs per replicate (i.e., 21 eggs per treatment) were randomly collected for egg quality evaluation. Eggshell thickness was measured at three locations (air cell, equator, and sharp end) using a micrometer caliper, and the mean value was recorded. Eggshell density (mg/cm²) was calculated by dividing eggshell weight by shell surface area. Shell surface area was estimated according to Mueller and Scott (1940), using the formula *S* = 4.47 × *W*²/³, where S is surface area and W is egg weight. Albumen height, Haugh unit, yolk colour, and shell strength were determined using a digital egg tester (DET-6000, Nabel Co., Ltd., Kyoto, Japan). Average egg weight was calculated weekly as the total egg weight divided by the number of eggs produced. Regarding the antioxidant analyses, fresh eggs (i.e., 21 eggs per treatment) were collected and egg yolk and egg white were separated, and additional eggs were stored at room temperature for 14 and 28 days, with storage temperature monitored daily. Lipid oxidation in yolk was assessed by determining malondialdehyde (**MDA**) concentration according to the method of Botsoglou et al. (2005). Moreover, protein oxidation in egg white was evaluated by measuring protein carbonyls following Min et al. (2005) and Liu et al. (2009).

### Tissue sampling, RNA isolation and reverse transcription to cDNA

Ovarian tissue sampling and RNA processing were performed following the general procedures described by Brouklogiannis et al. (2023). At the end of the 7th experimental week (layers; 57 weeks of age), 7 hens per treatment were randomly selected, anesthetized in accordance with EC 1099/2009, and euthanized by severing the jugular vein. The ovary was aseptically excised, snap-frozen in liquid nitrogen, and stored at −80°C until gene expression analysis. Total RNA was extracted from ovarian tissue using NucleoZOL Reagent (Macherey-Nagel, Düren, Germany) following the manufacturer’s instructions. RNA yield and purity were assessed with a NanoDrop-1000 spectrophotometer (Thermo Fisher Scientific, Waltham, UK). To remove residual genomic DNA, samples underwent DNase I treatment (New England Biolabs, Ipswich, UK) and were subsequently heat-inactivated in the presence of EDTA. RNA integrity was verified by agarose gel electrophoresis. Complementary DNA (**cDNA**) was synthesized from 500 ng total RNA using the PrimeScript RT Reagent Kit (Takara Bio Inc., Shiga-Ken, Japan), according to the manufacturer’s protocol, and stored at −20°C until quantitative PCR analysis.

### Quantitative real-time PCR analysis

The following *Gallus gallus* mRNA expression levels were examined: aryl hydrocarbon receptor 1 (***AhR1***), aryl hydrocarbon receptor nuclear translocator (***ARNT***), cytochrome P450 1A1 (***CYP1A1***), cytochrome P450 1B1 (***CYP1B1***), nuclear factor erythroid-derived 2-like 2 (**Nrf2**), kelch like ECH associated protein 1 (***KEAP1***), catalase (***CAT***), superoxide dismutase 1 (***SOD1***), glutathione peroxidase 2 (***GPX2***), glutathione-disulfide reductase (***GSR*)**, glutathione S-transferase alpha 2 (***GSTA2***), NAD(P)H quinone dehydrogenase 1 (***NQO1***), heme oxygenase 1 (***HMOX1***), toll-like receptor 2 family member B (***TLR2B***), toll like receptor 3 (***TLR3***), toll like receptor 4 (***TLR4***), myeloid differentiation primary response 88 (***MyD88***), interferon regulatory factor 3 (***IRF3***), toll like receptor adaptor molecule 1 (***TRIF***), nuclear factor kappa B subunit 1 (***NFκB1***), transforming growth factor beta 1 (***TGFB1***), interleukin 6 **(*IL6***), interferon-beta (***IFNW***), lipopolysaccharide induced TNF factor (***LITAF***), inducible nitric oxide synthase (***iNOS***) and cyclooxygenase 2 (***COX2***). Suitable primers were designed using the GenBank sequences deposited on the National Center for Biotechnology Information and US National Library of Medicine (**NCBI**) as shown in Table 2. Primers were checked using the PRIMER BLAST algorithm for *Gallus gallus* mRNA databases to ensure that there was a unique amplicon. Real-time quantitative PCR (qPCR) was accomplished in 96-well microplates with a SaCycler-96 Real-Time PCR System (Sacace Biotechnologies s.r.l., Como, Italy) and FastGene IC Green 2x qPCR universal mix (Nippon Genetics, Tokyo, Japan). Every reaction included 12.5 ng RNA equivalents along with 200 nmol/L of forward and reverse primers for each gene. The reactions were incubated at 95°C for 3 min, accompanied by 40 cycles of 95°C for 5 s, 59.5 to 62°C (depending on the target gene) for 20 s, 72°C for 33 s. This was tailed by a melt curve analysis to check the reaction specificity. Each sample was measured in duplicates. Relative expression ratios of target genes were calculated according to Pfaffl. (2001) and adapted for the multi-reference genes normalization procedure according to Hellemans et al. (2008) using glyceraldehyde 3-phosphate dehydrogenase (***GAPDH***) and beta (β)-actin (***ACTB***) as housekeeping genes.

**Table 2:** Oligonucleotide primers used for gene expression of selected targets by quantitative real-time PCR.

Gene^1^	Primer sequence (5′−3′)^2^	Annealing temperature (⁰C)	PCR product size (bp)	GenBank (NCBI Reference Sequence)
GAPDH	F: ACTTTGGCATTGTGGAGGGT R: GGACGCTGGGATGATGTTCT	59.5	131	NM_204305.1
ACTB	F: CACAGATCATGTTTGAGACCTT R: CATCACAATACCAGTGGTACG	60	101	NM_205518.1
Detoxification response				
AhR1	F: TTTAGTGTGGCAGGTGGATT R: CCTTGTGCCAATGATGCTATTTG	60	200	NM_204118.2
ARNT	F: GAGACCAAGGCCCCAACTAC R: TCGGGTGCCTCTTTCTTTCC	62	140	NM_204200.1
CYP1A1	F: GTGATGGAGGTGACCATCGG R: ACATTCGTAGCTGAACGCCA	62	165	NM_205147.1
CYP1B1	F: CAGTGACTCCGCATCCCAAA R: CCATACGCTTACGGCAGGTT	62	132	XM_015283751.2
Antioxidant response				
Nrf2	F: AGACGCTTTCTTCAGGGGTAG R: AAAAACTTCACGCCTTGCCC	60	285	NM_205117.1
Keap1	F: GGTTACGATGGGACGGATCA R: CACGTAGATCTTGCCCTGGT	62	135	XM_025145847.1
CAT	F: ACCAAGTACTGCAAGGCGAA R: TGAGGGTTCCTCTTCTGGCT	60	245	NM_001031215
SOD1	F: AGGGGGTCATCCACTTCC R: CCCATTTGTGTTGTCTCCAA	60	122	NM_205064.1
GPX2	F: GAGCCCAACTTCACCCTGTT R: CTTCAGGTAGGCGAAGACGG	62	75	NM_001277854.1
GSR	F: GTGGATCCCCACAACCATGT R: CAGACATCACCGATGGCGTA	62	80	XM_015276627.1
NQO1	F: GAGCGAAGTTCAGCCCAGT R: ATGGCGTGGTTGAAAGAGGT	60.5	150	NM_001277619.1
GSTA2	F: GCCTGACTTCAGTCCTTGGT R: CCACCGAATTGACTCCATCT	60	138	NM_001001776.1
HMOX1	F: ACACCCGCTATTTGGGAGAC R: GAACTTGGTGGCGTTGGAGA	62	134	NM_205344.1
Inflammatory response				
TLR2	F: CTTGGAGATCAGAGTTTGGA R: ATTTGGGAATTTGAGTGCTG	62	238	NM_001161650.1
TLR3	F: GCTTGGTTTGCTAGTTGGCT R: ACCGTGATATTTAGGCGGGG	59.5	93	NM_001011691.3
TLR4	F: GTCTCTCCTTCCTTACCTGCTGTTC R: AGGAGGAGAAAGACAGGGTAGGTG	64.5	187	NM_001030693.1
MYD88	F: AATGGACACTGAGCTCTGCC R: CAAACCCGATCTGTGGGACA	60	126	NM_001030962.3
IRF3	F: GAGGATCCGGCCAAATGGAA R: GCCAAATCGTGGTGGTTGAG	60	212	NM_205372.1
TRIF	F: TCAGCCATTCTCCGTCCTCTTC R: GGTCAGCAGAAGGATAAGGAAAGC	62	339	NM_001081506.1
NFκB1	F: GAAGGAATCGTACCGGGAACA R: CTCAGAGGGCCTTGTGACAGTAA	59	131	NM_205134.1
TGFB1	F: GGTTATATGGCCAACTTCTGCAT R: CCCCGGGTTGTGTTGGT	60	102	JQ423909.1
IL6	F: AAATCCCTCCTCGCCAATCT R: CCCTCACGGTCTTCTCCATAAA	59	106	NM_204628.1
IFNW	F: CCTCAACCAGATCCAGCATTAC R: CCCAGGTACAAGCACTGTAGTT	60.5	167	NM_001024836.1
LITAF	F: GAGCAGGGCTGACACGGAT R: GCACAAAAGAGCTGATGGCAG	60	149	NM_204267.1
iNOS	F: AAAGAAAGGGATCAAAGGTGGT R: CAAGCATCCTCTTCAAAGTCTG	60	296	NM_204961.1
COX2	F: CCGTTCCTCTACAACAACTCCA R: TTCCCACCAGAACCCTA	60	232	NM_001167719.2

1 GAPDH = glyceraldehyde 3-phosphate dehydrogenase; ACTB = actin beta; AhR1 = aryl hydrocarbon receptor 1; ARNT = aryl hydrocarbon receptor nuclear translocator; CYP1A1 = cytochrome P450 1A1; CYP1B1 = cytochrome P450 1B1; Nrf2 = nuclear factor; erythroid 2-like 2; Keap1 = kelch-like ECH-associated protein 1; CAT = catalase; SOD1 = superoxide dismutase 1; GPX2 = glutathione peroxidase 2; GSR = glutathione-disulfide reductase; GSTA2 = glutathione S-transferase alpha 2; NQO1 = NAD(P)H quinone dehydrogenase 1; HMOX1 = heme oxygenase 1; TLR2B = toll-like receptor 2B; TLR3 = toll-like receptor 3; TLR4 = toll-like receptor 4; TRIF = toll-like receptor adaptor molecule 1; MyD88 = myeloid differentiation primary response 88; IRF3 = interferon regulatory factor 3; NFκB1 = nuclear factor kappa B subunit 1; TGFB1 = transforming growth factor beta 1; IL6 = interleukin 6; IFNW = interferon beta; LITAF = lipopolysaccharide-induced TNF Factor; iNOS = nitric oxide synthase; COX2 = Cyclooxygenase 2.

2 F: Forward, R: Reverse.

### Statistical analysis

Experimental data on laying productive performance and total tract apparent digestibility of nutrients were analyzed on a cage basis, as each cage represented the experimental unit. Egg quality traits and egg antioxidant indices were analyzed on a replicate basis, using data obtained from three eggs per replicate pooled and homogenized prior to analysis. Data on ovarian mRNA expression levels were analyzed on an individual hen basis. All data were initially checked for normality using the Kolmogorov-Smirnov test and subsequently analyzed with the general linear model (GLM) - ANOVA procedure using the SPSS for Windows statistical package program, version 27 (SPSS Inc., Chicago, IL). Yolk colour was analyzed with the Kruskal–Wallis non-parametric one-way ANOVA. Statistically significant effects were further analyzed, and means were compared using Tukey's honest significant difference (**HSD**) multiple comparison procedure. The level of significance was set at *P* ≤ 0.05. Linear (**lin**) and quadratic (**quad**) response patterns to the dietary spent mushroom substrate inclusion level were studied using polynomial contrasts.

## Results

### Laying productive performance

The effects of the spent mushroom substrate (SMS) supplementation on the overall productive performance responses of laying hens are shown in Table 3. Compared to the control treatment, dietary supplementation of SMS led to significant (*P* ≤ 0.05) differences in laying rate and egg mass for the overall period (1-7 experimental weeks, i.e., 51-57 weeks of layers age). In detail, hens fed the highest SMS level (S100) showed the greatest improvements in laying rate and egg mass, increasing from 92.8% to 95.7% and from 59.3 to 61.8 g/hen/day compared with the control. There were no (*P* > 0.05) differences between the experimental treatments for the overall feed intake and feed conversion ratio. Polynomial contrast analysis revealed that overall laying rate, egg mass and feed intake increased in a linear (*P_lin_* = 0.003, 0.001 and 0.018, respectively) manner with increasing SMS supplementation. Mortality throughout the experiment was zero.

**Table 3:** Effects of dietary spent mushroom substrate on the overall laying productive performance.

Responses		Treatments^1^					Statistics^2^		
(1 to 7 week^4^)		S0	S25	S50	S100	SEM^3^	*P_anova_*	*P_linear_*	*P_quadratic_*
Laying Rate %		92.8^b^	94.6^ab^	95.2^ab^	95.7^a^	0.89	0.018	0.003	0.252
Egg mass *(*g/hen/d)		59.3^B^	60.6^AB^	61.2^AB^	61.8^A^	0.68	0.009	0.001	0.489
Feed intake (g/hen/d)		114.1	114.9	116.3	118.3	1.73	0.110	0.018	0.603
Feed conversion ratio		1.93	1.90	1.90	1.91	0.024	0.624	0.780	0.235

1 SMS supplementation (S0=0 g/kg, S25=25 g/kg, S50=50 g/kg and S100=100 g SMS/kg of diet, respectively). Data represent treatment means based on 7 hens per replicate and 7 replicates per treatment (*n* = 7).

2 Means with no common superscripts (a, b or A, B) within the same row differ significantly (*P* ≤ 0.05 or 0.01).

3 Standard error of the means.

4 Reference to 1-7 weeks of the overall experimental period corresponds to 51-57 weeks of the layers' age.

### Total tract apparent digestibility of nutrients

The effects of spent mushroom substrate (SMS) supplementation on total tract apparent digestibility coefficients of nutrients in laying hens’ diets are shown in Table 4. Dietary SMS supplementation affected nutrient digestibility coefficients of ether extract. In detail, ether extract digestibility was affected by SMS supplementation (*P* = 0.002), exhibiting both linear (*P_lin_* = 0.003) and quadratic (*P_quad_* = 0.007) pattern of increase, and was higher in all SMS-supplemented treatments compared with the S0. Moreover, dry matter, ash and crude protein digestibility did not differ between the experimental treatments (*P* > 0.05).

**Table 4:** Effects of dietary spent mushroom substrate on total tract apparent digestibility coefficients of nutrients in laying hens.

Components		Treatments^1^					Statistics^2^		
		S0	S25	S50	S100	SEM^3^	*P_anova_*	*P_linear_*	*P_quadratic_*
Dry matter		73.02	74.80	75.28	74.08	1.065	0.191	0.288	0.058
Ash		58.75	57.73	55.97	55.40	2.255	0.261	0.210	0.226
Ether extract		89.98^B^	92.38^A^	93.67^A^	92.54^A^	0.848	0.002	0.003	0.007
Crude protein		68.74	69.63	68.38	69.30	2.841	0.972	0.964	0.994

1 SMS supplementation (S0=0 g/kg, S25=25 g/kg, S50=50 g/kg and S100=100 g SMS/kg of diet, respectively). Data represent treatment means based on 7 cage replicates per treatment (*n* = 7).

2 Means with no common superscripts (A, B) within the same row differ significantly (*P* ≤ 0.01).

3 Standard error of the means.

### Egg quality traits

Egg quality traits of laying hens fed SMS-supplemented diets are presented in Table 5. Dietary SMS supplementation had no effects (*P* > 0.05) on egg weight, shell mass, shell thickness, shell density, albumen height, Haugh unit and yolk colour. However, polynomial contrast analysis revealed linear pattern of increase (*P_lin_* < 0.043) in shell breaking strength, with the highest value noted in the S100 treatment.

**Table 5:** Effects of dietary spent mushroom substrate on egg quality traits of laying hens.

Egg quality traits		Treatments^1^					Statistics^2^		
(week^4^ 7)		S0	S25	S50	S100	SEM^3^	*P_anova_*	*P_linear_*	*P_quadratic_*
Egg weight (g)		63.5	63.1	63.9	63.9	0.73	0.700	0.426	0.753
Shell mass (g)		6.165	6.100	6.135	6.145	0.192	0.704	0.801	0.705
Shell thickness (mm)		0.385	0.372	0.387	0.379	0.011	0.535	0.955	0.785
Shell density (mg/cm^2^)		83.95	82.86	83.88	82.44	2.382	0.895	0.644	0.915
Shell strength (kgf)		4.45	4.71	4.84	5.13	0.317	0.226	0.043	0.945
Albumen height (mm)		6.8	7.1	6.9	7.0	0.37	0.845	0.746	0.936
Haugh unit		81.5	82.8	80.6	82.3	2.44	0.810	0.984	0.931
Yolk colour^5^		6.1	5.9	6.0	6.0	0.14	0.277	0.533	0.170

1 SMS supplementation (S0=0 g/kg, S25=25 g/kg, S50=50 g/kg and S100=100 g SMS/kg of diet, respectively). Data represent treatment means based on 3 eggs per replicate and 7 replicates per treatment (*n* = 7).

2 The level of significance was set at *P* ≤ 0.05.

3 Standard error of the means.

4 Reference to week 7 of the experimental period corresponds to the 57th week of the layers' age.

5 Yolk colour (expressed in YolkFan™ units, 1–16) was analyzed with the Kruskal–Wallis non-parametric one-way ANOVA.

### Antioxidant capacity of hen eggs

Dietary SMS supplementation affected (*P* ≤ 0.05) antioxidant markers in both fresh and stored eggs as shown in Table 6. In detail, fresh egg yolks (0 d) from the S100 treatment had the lowest (*P* < 0.001) MDA concentrations, with intermediate values in S25 and S50, compared to S0. Similar treatment effects were observed after 14 and 28 days of storage, where S50 and S100 (*P* < 0.001) had lower MDA levels, always compared to the control treatment. Increasing SMS inclusion level resulted in linear pattern of decrease in yolk MDA at 0, 14, and 28 days of storage (Pₗᵢₙ < 0.001, 0.035, and < 0.001, respectively). Carbonyl content in egg white was also affected by SMS supplementation at 0 and 28 days (*P* ≤ 0.001), with lower protein oxidation levels observed primarily in S100 and secondarily in S50 compared to S0. At 14 days of storage, carbonyl content tended to be lower (*P* = 0.081) in the SMS treatments compared with the control. Polynomial contrast analysis revealed patterns of egg white carbonyl content decrease (Pₗᵢₙ ≤ 0.05) in a linear manner at all sampling points (i.e. 0, 14 and 28 days of storage).

**Table 6:** Effects of dietary spent mushroom substrate on the antioxidant capacity of fresh and stored eggs from laying hens.

Item	Days of storage		Treatments^1^					Statistics^2^		
			S0	S25	S50	S100	SEM^3^	*P_anova_*	*P_linear_*	*P_quadratic_*
MDA-yolk (μg MDA/ g egg yolk)	0d (fresh)	2.81^A^		1.78^B^	1.35^BC^	1.00^C^	0.237	<0.001	<0.001	0.338
	14d	3.48^A^		3.10^AB^	2.56^BC^	2.27^C^	0.271	<0.001	0.035	0.151
	28d	4.56^A^		4.14^AB^	3.76^BC^	3.23^C^	0.267	<0.001	<0.001	0.469
Carbonyl S0tent- white (nmol DNPH/mg protein)	0d (fresh)	1.45^A^		1.29^AB^	1.14^BC^	0.98^C^	0.231	<0.001	<0.001	0.637
	14d	1.65		1.37	1.28	1.09	0.253	0.081	0.05	0.963
	28d	2.16^A^		1.82^BC^	1.64^C^	1.57^C^	0.150	0.001	<0.001	0.924

^1^SMS supplementation (S0=0 g/kg, S25=25 g/kg, S50=50 g/kg and S100=100 g SMS/kg of diet, respectively). Data represent treatment means based on 3 eggs per replicate and 7 replicates per treatment (*n* = 7).

^2^Means with different superscripts (a, b, c or A, B, C) within the same row differ significantly (*P* ≤ 0.05 or 0.01).

^3^Standard error of the means.

^4^Reference to week 7 of the experimental period corresponds to the 57th week of the layers' age.

### Relative expression of the detoxification response-related genes in the ovary

In the ovary, the relative expression levels of the detoxification response-related genes (AhR pathway: *AhR1, ARNT, CYP1A1 and CYP1B1*) at week 7 (i.e., 57 week of layers age) are presented in Table 7*.* The expression of *AhR1* (*P* = 0.043), *ARNT* (*P* = 0.031) and *CYP1A1* (*P* < 0.00) differed between the experimental treatments. Polynomial contrast analysis revealed that increasing SMS supplementation level resulted in linear patterns of decrease for *AhR1* (*P_lin_* = 0.007)*, ARNT* (*P_lin_* = 0.005) and *CYP1A1*(*P_lin_* < 0.001). The relative gene expression of *AhR1, ARNT* and *CYP1A1* was lowest primarily in treatment S100 and secondarily in S50, compared to the control treatment S0.

**Table 7:** Relative gene expression levels of the detoxification response-related genes in layers’ ovary.

Ovary	AhR pathway		Treatments^1^					Statistics^2^		
	Genes^4^		S0	S25	S50	S100	SEM^3^	*P_anova_*	*P_linear_*	*P_quadratic_*
week 7 (57-wks old)	AhR1	1.39^a^		1.09^ab^	0.83^b^	0.80^b^	0.220	0.043	0.007	0.380
	ARNT	1.45^a^		1.10^ab^	0.81^ab^	0.79^b^	0.233	0.031	0.005	0.334
	CYP1A1	1.45^A^		0.97^B^	1.01^B^	0.49^C^	0.151	<0.001	<0.001	0.811
	CYP1B1	1.13		1.27	0.82	1.01	0.197	0.160	0.207	0.891

1 SMS supplementation (S0=0 g/kg, S25=25 g/kg, S50=50 g/kg and S100=100 g SMS/kg of diet, respectively). Data represent treatment means based on 1 ovary sample per replicate and 7 replicates per treatment (*n* = 7).

2 Means with different superscripts (a, b, c or A, B, C) within the same row differ significantly (*P* ≤ 0.05 or 0.01).

3 Standard error of the means.

4 AhR1 = aryl hydrocarbon receptor 1; ARNT = aryl hydrocarbon receptor nuclear translocator; CYP1A1 = cytochrome P450 1A1; CYP1B1 = cytochrome P450 1B1.

### Relative expression of the antioxidant response-related genes in the ovary

The relative expression levels of the antioxidant response-related genes (Nrf2 pathway: *Nrf2, KEAP1, CAT, SOD1, GPX2, GSR, GSTA2, NQO1* and *HMOX1*) at week 7 (i.e., 57 week of layers age) are presented in Table 8. At the ovarian level, the SMS supplementation up-regulated the relative expression of *Nrf2* (*P* = 0.007), *CAT* (*P* = 0.001), *SOD1* (*P* = 0.021), *GSTA2* (*P* < 0.033) and *HMOX1* (*P* = 0.002), compared to S0. Increasing SMS inclusion level resulted in linear patterns of increase for *Nrf2* (*P_lin_* = 0.001), *CAT* (*P_lin_* < 0.001), *SOD1* (*P_lin_* = 0.005), *GSTA2* (*P_lin_* = 0.004) and *HMOX1* (*P_lin_* = 0.013). In the case of *Nrf2, CAT, SOD1, GSTA2* and *HMOX1* the S100 treatment had the highest expression, compared to the S0.

**Table 8:** Relative gene expression levels of the antioxidant response-related genes in layers’ ovary.

Ovary	Nrf2 pathway		Treatments^1^					Statistics^2^		
	Genes^4^		S0	S25	S50	S100	SEM^3^	*P_anova_*	*P_linear_*	*P_quadratic_*
week 7 (57-wks old)	Nrf2	0.81^B^		1.29^AB^	1.22^AB^	1.69^B^	0.225	0.007	0.001	0.968
	KEAP1	1.14		1.03	1.04	1.04	0.261	0.970	0.725	0.762
	CAT	0.70^B^		1.22^AB^	1.41^A^	1.61^A^	0.227	0.001	<0.001	0.335
	SOD1	0.93^a^		1.25^ab^	1.20^ab^	1.71^b^	0.232	0.021	0.005	0.563
	GPX2	0.99		1.12	1.10	1.42	0.260	0.413	0.136	0.613
	GSR	1.10		1.44	1.41	1.35	0.161	0.165	0.165	0.094
	GSTA2	0.88^a^		1.11^ab^	1.25^ab^	1.59^b^	0.229	0.033	0.004	0.740
	NQO1	1.03		1.20	1.03	1.39	0.289	0.566	0.333	0.653
	HMOX1	0.74^B^		1.17^AB^	1.04^AB^	1.28^A^	0.177	0.002	0.013	0.441

1 SMS supplementation (S0=0 g/kg, S25=25 g/kg, S50=50 g/kg and S100=100 g SMS/kg of diet, respectively). Data represent treatment means based on 1 ovary sample per replicate and 7 replicates per treatment (*n* = 7).

2 Means with different superscripts (a, b or A, B) within the same row differ significantly (*P* ≤ 0.05 or 0.01).

3 Standard error of the means.

4 Nrf2 = nuclear factor; erythroid 2-like 2; Keap1 = kelch-like ECH-associated protein 1; CAT = catalase; SOD1 = superoxide dismutase 1; GPX2 = glutathione peroxidase 2; GSR = glutathione-disulfide reductase; GSTA2 = glutathione S-transferase alpha 2; NQO1 = NAD(P)H quinone dehydrogenase 1; HMOX1 = heme oxygenase 1.

### Relative expression of the inflammatory response-related genes in the ovary

The relative expression levels of the inflammatory response-related genes (TLR signaling to NF-κB pathway: *TLR2B, TLR3, TLR4, MyD88, IRF3, TRIF, NFκB1, TGFB1, IL6, IFNW, LITAF, iNOS* and *COX2*) at week 7 (i.e., 57 week of layers age) are presented in Table 9. The dietary SMS inclusion down-regulated the expression of *MYD88* (*P* < 0.001), *NFκB1* (*P* = 0.001), *TGFB1* (*P* = 0.001), *IL6* (*P* = 0.004), *IFNW* (*P* < 0.001), *LITAF* (*P* = 0.026), *iNOS* (*P* = 0.010) and *COX2* (*P* = 0.024), compared to S0. Polynomial contrast analysis showed that the relative expression of *TLR2B* (*P_lin_* = 0.019)*, MyD88* (*P_lin_* = 0.008)*, TRIF* (*P_lin_* = 0.014)*, NFκB1* (*P_lin_* < 0.001)*, TGFB1* (*P_lin_* = 0.008)*, IL6* (*P_lin_* = 0.001)*, IFNW* (*P_lin_* = 0.046)*, LITAF* (*P_lin_* = 0.005)*, iNOS* (*P_lin_* = 0.003) and *COX2* (*P_lin_* = 0.006) displayed linear patterns of decrease. Compared to the S0, the relative expression levels were lowest for S50 and S100 for the first (i.e., *NFκΒ1, IL6*, iNOS and *COX2*) and second (i.e., *MYD88, TGFB1, IFNW* and *LITAF*) set of genes above, respectively.

**Table 9:** Relative gene expression of the inflammatory response-related genes in layers’ ovary.

Ovary	TLR to NF-κB		Treatments^1^					Statistics^2^		
	Genes^4^		S0	S25	S50	S100	SEM^3^	*P_anova_*	*P_linear_*	*P_quadratic_*
week 7 (57-wks old)	TLR2	1.12		1.10	0.76	0.63	0.225	0.098	0.019	0.739
	TLR3	1.20		1.28	0.90	0.95	0.187	0.151	0.071	0.881
	TLR4	0.89		1.13	0.87	0.68	0.189	0.164	0.159	0.119
	MYD88	1.19^A^		1.09^A^	0.79^AB^	0.61^B^	0.223	<0.001	0.008	0.812
	IRF3	0.93		0.96	0.96	0.61	0.211	0.289	0.164	0.208
	TRIF	1.09		1.23	0.94	0.97	0.208	0.195	0.014	0.483
	NFκB1	1.25^A^		1.32^A^	0.64^B^	0.72^B^	0.173	0.001	<0.001	0.963
	TGFB1	0.83^A^		0.98^A^	0.97^A^	0.40^B^	0.144	0.001	0.008	0.364
	IL6	1.39^A^		1.22^AB^	0.69^C^	0.92^BC^	0.156	0.004	0.001	0.081
	IFNW	1.09^A^		1.06^A^	0.92^AB^	0.44^B^	0.213	<0.001	0.046	0.115
	LITAF	1.56^a^		1.07^ab^	0.70^b^	0.69^b^	0.303	0.026	0.005	0.274
	iNOS	1.11^A^		1.05^AB^	0.54^B^	0.59^B^	0.194	0.010	0.003	0.696
	COX2	1.47^a^		1.13^ab^	0.51^b^	0.64^ab^	0.324	0.024	0.006	0.307

1 SMS supplementation (S0=0 g/kg, S25=25 g/kg, S50=50 g/kg and S100=100 g SMS/kg of diet, respectively). Data represent treatment means based on 1 ovary sample per replicate and 7 replicates per treatment (*n* = 7).

2 Means with different superscripts (a, b, c or A, B, C) within the same row differ significantly (*P* ≤ 0.05 or 0.01).

3 Standard error of the means.

4 TLR2, 3, 4 = toll-like receptor 2, 3, 4; MyD88 = myeloid differentiation primary response 88; IRF3 = interferon regulatory factor 3; TRIF = toll-like receptor adaptor molecule 1; NFκB1 = nuclear factor kappa B subunit 1; TGFB1 = transforming growth factor beta 1; IL6 = interleukin 6; IFNW = interferon-beta; LITAF = lipopolysaccharide-induced TNF factor; iNOS = inducible nitric oxide synthase; COX2 = Cyclooxygenase 2.

## Discussion

This study, conducted under non-challenge conditions, evaluated the effects of dietary *Pleurotus ostreatus* spent mushroom substrate (SMS) on productive performance, nutrient digestibility, egg quality traits, and oxidative stability, while it aimed to generate baseline physiological information on ovarian AhR/Nrf2/NF-κB pathway components in mid-phase laying hens. This work is particularly relevant given the scarcity of data on dietary SMS use in layers and the increasing interest in valorizing mushroom-derived by-products within animal nutrition. SMS produced from *Pleurotus* spp. cultivation is more than just a fibrous residue; it is increasingly recognized as a source of bioactive compounds, including phenolics, ergosterol, β-glucans, and other fungal cell-wall components (Bederska-Łojewska et al., 2017; Martín et al., 2023; Törős et al., 2024). These compounds possess cytoprotective, antimicrobial, and anti-inflammatory properties, making them beneficial for animal performance, egg quality, resilience and health (Hwang, 2012; Natsir and Wicaksono, 2020; Ogbe et al., 2009; Törős et al., 2024). Beyond its bioactive profile, the dietary use of SMS contributes to the sustainable valorization of residues generated by the mushroom industry.

In this study, the feeding trial began when hens were in the mid-lay phase (i.e., layers 50 weeks of age) and continued for 7 weeks. SMS was evaluated from the perspective of its bioactive compound profile; therefore, all diets were formulated to be isocaloric, isonitrogenous, and isofibrous, with wheat straw included in the control diet (S0) and proportionally replaced by SMS in the supplemented treatments. Despite the elevated dietary fiber content (6.8-7%) across all treatments, including the control, hens were healthy throughout the trial, and the productive performance of the control group were well in line the respective performance benchmarks reported in the Hy-Line Brown Management Guide 2018. This confirms that the dietary fiber level used in this study did not compromise bird welfare or baseline productivity. The zootechnical performance results demonstrated that dietary SMS supplementation improved overall laying rate and egg mass in a dose-dependent manner. Hens fed the highest SMS inclusion (S100) exhibited a laying rate that was approximately 3% higher than the control treatment, with concurrent increases in egg mass. These enhancements may collectively reflect the contribution of SMS-derived bioactive compounds which are known to exert health-promoting effects, thereby supporting reproductive function and overall productivity. Furthermore, the noted effects on nutrients digestibility further support the performance improvements. While dry matter, ash and crude protein digestibility remained unaffected, apparent digestibility of ether extract and ash improved in SMS-fed hens. Enhanced lipid utilization is noteworthy because *Pleurotus*-derived residues contain fungal cell-wall components such as β-glucans and chitinous material, which may modulate gut microbial activity and digestive enzyme secretion (Bekiaris et al., 2020; Mateos et al., 2012; Singh and Kim, 2021; Törős et al., 2024).

Inclusion of SMS at up to 100 g/kg diet did not significantly affect the internal and external egg quality traits, except for a linear pattern of improvement in shell breaking strength with increasing SMS inclusion level. The egg quality traits were preserved across treatments, indicating that the SMS inclusion did not disrupt structural or functional egg characteristics. More pronounced responses emerged in oxidative stability, where SMS supplementation reduced yolk MDA concentrations and egg-white carbonyls in both fresh and stored eggs up to 28 days. These improvements suggest that the antioxidant potential of SMS, attributed to its antioxidant compounds such as phenolics, conferred greater resilience of lipids and proteins to oxidative deterioration (Koutrotsios et al., 2017; Törős et al., 2024). Notably, the protective effects were maintained throughout storage, highlighting that SMS not only supported the production of eggs with stable physical attributes, but also enhanced their biochemical robustness by limiting oxidative damage during shelf-life. Overall, SMS supplementation improved the oxidative stability of egg yolk and white in a dose-dependent manner during storage.

The ovary is a critical reproductive organ that is particularly vulnerable to oxidative damage, a susceptibility that becomes more pronounced as hens progress in age and reproductive intensity. Ovarian dysfunction and age-related decline could adversely affect poultry performance, reproductive capacity, hatchability, egg quality and life span (Brouklogiannis and Mountzouris, 2025; Valdez and Petroff, 2004; Zhang et al., 2022). In order to investigate how dietary SMS may modulate the molecular systems that preserve ovarian homeostasis, we examined its effects on gene expression across three key regulatory pathways, which collectively orchestrate cytoprotective and inflammatory responses. To our knowledge, this is the first study to map these pathway-specific effects of SMS in the ovarian tissue of laying hens. The major homeostasis-related pathways examined in this study include the aryl hydrocarbon receptor (AhR), which regulates detoxification processes; the nuclear factor erythroid 2–related factor 2 (Nrf2), a key mediator of adaptive antioxidant defense; and the nuclear factor kappa B (NF-κB), which regulates immune and inflammatory signaling cascades (Morgan and Liu, 2011; Mountzouris and Brouklogiannis, 2024).

The AhR functions as a ligand-activated transcription factor that responds to a range of endogenous and dietary compounds, including xenobiotics and various bioactive molecules (Anagnostopoulos et al., 2023; Antos et al., 2015; Mountzouris and Brouklogiannis, 2024). Upon activation, it regulates genes such as *ARNT, CYP1A1*, and *CYP1B1* that are involved in detoxification processes, helping cells adapt to chemical or oxidative challenges. Beyond xenobiotic metabolism, AhR also intersects with antioxidant and inflammatory-related pathways, positioning it as an important coordinator of cellular homeostasis (Brouklogiannis et al., 2025; Larigot et al., 2018). In addition to the improvements in performance, nutrient digestibility, and egg oxidative stability, SMS supplementation also influenced the ovarian AhR pathway genes assessed. At the 7th week of the trial (layers 57 weeks of age), most of the AhR-related genes (3 of 4) were down-regulated in hens receiving SMS. Such mRNA decreased levels may reflect lower requirements for detoxification in the ovary. Although AhR is classically linked to xenobiotic metabolism, it also participates in reproductive regulation, and excessive activation has been associated with accelerated ovarian aging (Valdez and Petroff, 2004). Therefore, the dietary SMS-related down-regulation of AhR pathway genes may indicate a shift toward a more favorable cytoprotective environment in the ovary.

The Nrf2 pathway serves as a central defense system against oxidative damage and stress. Under non-challenge conditions, Nrf2 is restrained by its repressor kelch-like ECH-associated protein 1 (*Keap1*) (Ahmed et al., 2017; Vomund et al., 2017). Upon exposure to bioactive compounds, it translocates to the nucleus and induces a suite of cytoprotective genes, namely phase II enzymes involved in antioxidant defense (e.g., *CAT, SOD1, GPX2, GSR*), detoxification processes (e.g., *GSTA2, NQO1*), and anti-inflammatory activity (e.g., *HMOX1*) (Ahmed et al., 2017; Mountzouris and Brouklogiannis, 2024). In this study, dietary SMS enhanced the expression of Nrf2 and several of its downstream target genes in the ovary. Notably, antioxidant priming was evident, as 5 of the 9 Nrf2-related genes assessed were beneficially modulated in SMS-fed hens. Taken together, the ovarian transcriptomic findings indicate that SMS supplementation enhances the birds’ adaptive antioxidant capacity against potential oxidative challenges. Oxidative stress is a key driver of ovarian aging and related functional decline, and maintaining effective control of ROS is essential for sustaining reproductive performance and overall health (Durand et al., 2022; Zhang et al., 2022). The observed up-regulation of Nrf2-associated genes therefore suggests that SMS may contribute strengthen the antioxidant buffering reservoir needed to preserve ovarian homeostasis during mid-lay period.

Widely regarded as the central regulator of inflammatory signaling, the NF-κB is activated by a wide array of stimuli, including cytokines, stressor challenges, and microbial components sensed among others through the Toll-like receptors (TLRs) (Ahmed et al., 2017; Paraskeuas and Mountzouris, 2019). Once triggered, the NF-κB regulates the expression of pro-inflammatory mediators such as *IL1B, TNF*, and *IL6*, along with chemokines and other downstream components (e.g., *iNOS, COX2*) that coordinate innate immune responses (Brouklogiannis and Mountzouris, 2025; Kogut et al., 2018). Although inflammation is essential for maintaining host defense, sustained or excessive inflammatory response could impose a considerable metabolic burden and disrupt tissue function, underscoring the importance of its balanced regulation for preserving ovarian homeostasis and supporting optimal host’s performance (Kogut et al., 2018; Lauridsen, 2019; Mountzouris and Brouklogiannis, 2024). In this study, the dietary SMS also showed immunomodulatory functions through its influence on TLR signaling to NFκB pathway. Particularly, dietary SMS inclusion led to the down-regulation of the majority of the inflammatory genes assessed in the ovary, with 8 of the 13 target genes showing reduced expression. The nutrigenomic results observed in this work may be linked to the capacity of SMS-derived β-glucans to influence inflammatory signaling, either by modulating TLRs or the transcription factor NFκB, as previously demonstrated for other bioactive polysaccharides (Cox and Dalloul, 2010; Goodridge et al., 2009; Jacob and Pescatore, 2017). Accordingly, our transcriptomic findings indicate that SMS supplementation helps maintain inflammatory responses at a level that supports ovarian cellular homeostasis, thereby contributing to improved resilience and sustained performance in laying hens.

Taking together, the molecular responses observed in the ovary provide mechanistic insights for the overall layer performance and egg quality. The dietary SMS introduced a fungal-derived mixture of bioactive compounds that broadened the spectrum of nutrigenomic mechanisms capable of fostering cellular homeostasis, health, and productivity. Notably, the most pronounced effects were detected in hens receiving the highest SMS inclusion level (100 g/kg), indicating a dose-responsive modulation of genes associated with cytoprotection and management of inflammation. Collectively, these findings demonstrate that a by-product of mushroom cultivation could induce biologically meaningful adjustments in metabolic pathways, thereby expanding current understanding of alternative feeding strategies in poultry nutrition. However, the study results support the potential of SMS inclusion in layers’ diets during the mid-laying phase. In order to recommend an overall inclusion of SMS in layers nutrition, further studies and analytical approaches would be necessary. In this sense, SMS application should be studied throughout the lifespan of the hens. In addition to the nutrigenomics responses studied in this work, ovarian histological and histopathological evaluation, as well as intestinal morphometric analysis and assessment of nutrient transporter expression would be of essential value.

In conclusion, the findings of this study provide evidence that dietary inclusion of *Pleurotus ostreatus* spent mushroom substrate (SMS) improved laying performance, nutrient digestibility, and egg oxidative stability in mid-phase hens. In addition, the ovarian nutrigenomic outcome provides a new mechanistic insight, showing that dietary SMS beneficially modulates key cytoprotective and inflammation-related genes, with the strongest effects observed at 100 g/kg. These findings suggest that SMS supports cellular homeostasis and physiological resilience while offering a sustainable approach to valorizing mushroom industry by-products. Further work including the evaluation of SMS inclusion along the hens’ lifespan complemented with further analytical techniques is warranted to evaluate its long-term applicability and to clarify its mechanisms across different laying stages and under potential stressor challenges.

## Funding

This research was carried at the Laboratory of Nutritional Physiology and Feeding of the Agricultural University of Athens and did not receive any specific grant from funding agencies in the public, commercial, or not-for-profit sectors.

## CRediT authorship contribution statement

**Ioannis Brouklogiannis:** Writing – original draft, Investigation, Formal analysis, Conceptualization. **Georgios Koutrotsios:** Writing – review & editing, Investigation. **Georgios I. Zervakis:** Writing – review & editing, Investigation. **Konstantinos C. Mountzouris:** Writing – review & editing, Supervision, Project administration, Investigation, Conceptualization.

## Disclosures

The authors declare that they have no known competing financial interests or personal relationships that could have appeared to influence the work reported in this paper.
